# Dietary Hyaluronic Acid Migrates into the Skin of Rats

**DOI:** 10.1155/2014/378024

**Published:** 2014-10-14

**Authors:** Mariko Oe, Koichi Mitsugi, Wataru Odanaka, Hideto Yoshida, Ryosuke Matsuoka, Satoshi Seino, Tomoyuki Kanemitsu, Yasunobu Masuda

**Affiliations:** ^1^R&D Division, Kewpie Corporation, 2-5-7 Sengawa Kewport, Sengawa, Chofu, Tokyo 182-0002, Japan; ^2^ADME & Tox. Research Institute, Sekisui Medical Co., Ltd., 2117 Muramatsu, Tokai-mura, Naka-gun, Ibaraki 319-1182, Japan

## Abstract

Hyaluronic acid is a constituent of the skin and helps to maintain hydration. The oral intake of hyaluronic acid increases water in the horny layer as demonstrated by human trials, but in vivo kinetics has not been shown. This study confirmed the absorption, migration, and excretion of ^14^C-labeled hyaluronic acid (^14^C-hyaluronic acid). ^14^C-hyaluronic acid was orally or intravenously administered to male SD rats aged 7 to 8 weeks. Plasma radioactivity after oral administration showed the highest level 8 hours after administration, and orally administered ^14^C-hyaluronic acid was found in the blood. Approximately 90% of ^14^C-hyaluronic acid was absorbed from the digestive tract and used as an energy source or a structural constituent of tissues based on tests of the urine, feces, expired air, and cadaver up to 168 hours (one week) after administration. The autoradiographic results suggested that radioactivity was distributed systematically and then reduced over time. The radioactivity was higher in the skin than in the blood at 24 and 96 hours after administration. The results show the possibility that orally administered hyaluronic acid migrated into the skin. No excessive accumulation was observed and more than 90% of the hyaluronic acid was excreted in expired air or urine.

## 1. Introduction

Hyaluronic acid is a high molecular mass linear polysaccharide composed of D-glucuronic acid and N-acetyl-D-glucosamine [1]. Hyaluronic acid is well known and was first isolated and identified from cattle eyes by Meyer and Palmer in 1934 [2]. Hyaluronic acid is synthesized by all animals and in some microbes, existing in all connective tissues of the body, including the skin, joint fluid, blood vessels, serum, brain, cartilage, heart valves, and umbilical cord. The total volume of hyaluronic acid in the body is about 15 g for an adult weighing 70 kg, replacing one third of the hyaluronic acid after degradation and synthesis everyday [3].

Hyaluronic acid is used in medicines, cosmetics, and foods and is a material receiving attention worldwide. Mixed into supplements, confectioneries, beverages, and processed foods, hyaluronic acid is approved as health food material for new resource foods in China, food additives and health function food in Korea, and as a food additive in Japan. Hyaluronic acid is marketed as a supplement in the USA, Canada, Italy, and Belgium.

Safety tests of hyaluronic acid included repeated dose oral toxicity tests [4], chronic toxicity tests [5], acute toxicity tests [6–8], subacute toxicity tests [9–11], reproductive and developmental toxicity studies [12–18], antigenicity tests [19, 20], mutagenicity tests [21–23], and micronucleus assays [24, 25]. Safety was confirmed; hence, hyaluronic acid is a food ingredient that can be ingested with confidence.

Hyaluronic acid supplements are used to treat joint pain in Europe and America, whereas it is known as a moisturizer for the skin in addition to treating joint pain in Japan. The oral intake of hyaluronic acid is reported to increase water in the horny layer as demonstrated in human trials [26–28]. On the other hand, further study is required whether orally administered high-molecular hyaluronic acid is taken up into the body and exerts effects.

Starch, a high molecular polysaccharide, is degraded into disaccharides by saliva and pancreatic juice and further degraded into monosaccharides to be absorbed by the small-intestinal epithelial cells in the small intestine.

Orally ingested fiber, such as cellulose, was not typically considered to be absorbed into the body but this fiber was reported to ferment in the presence of intestinal bacteria and was then taken up by the body [29]. In contrast, studies using Caco-2 cells reported that hyaluronic acid permeated the intestinal epithelium while retaining the chemical structure [30], and hyaluronic acid was taken up into the body via oral administration [31, 32] but the study results were few in number, and thus there was controversy over in vivo absorption. In articles that investigated in vivo absorption of hyaluronic acid [31, 32], a complex of radiolabeled technetium and hyaluronic acid was used via the oral route; therefore, the absorption results of technetium-hyaluronic acid molecular architecture may not be obtained due to separation of the complex or progression of ligand substitution reaction between the complex and the biological constituents [33].

Orally administered hyaluronic acid migrates into connective tissues, such as skin [31, 32], but metabolism and excretion after migration into the connective tissue have not been adequately examined.

In order to confirm the series of biokinetic processes of hyaluronic acid from absorption to excretion, we measured the plasma radioactivity level; excretion rate in urine, feces, and expired air; the residual rate in the body; and examined ^14^C distribution in each tissue with whole body autoradiography for single oral or single intravenous administration in rats using ^14^C-hyaluronic acid, a stable molecular architecture with ^14^C incorporated into the carbon skeleton.

## 2. Materials and Methods

### 2.1. Radiolabeled Compounds

**Figure 1:**
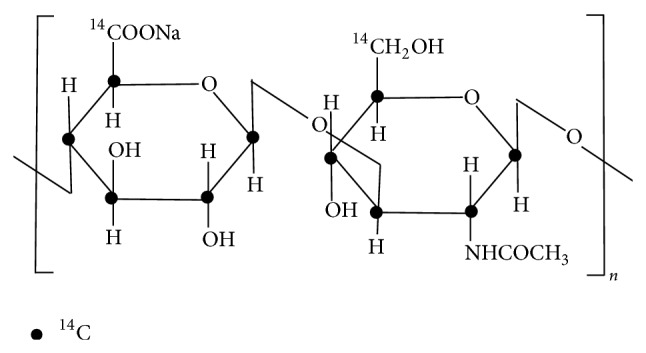
Structure of ^14^C hyaluronic acid.

We synthesized ^14^C-hyaluronic acid based on the tissue culture method (^14^C glucose was added in the culture medium) using the crista galli in the ADME/TOX Research Institute, Daiichi Pure Chemicals Co., Ltd., Ibaraki Prefecture, Japan. ^14^C-hyaluronic acid (Figure 1) was obtained by vacuum drying after purifying with hydrous ethanol. The average molecular weight was 920,000 from measuring with the limiting viscosity method. Specific radioactivity was 81.7 kBq/mg, and radiochemical purity was 98.6%.

### 2.2. Laboratory Animals

We purchased male SD rats aged 7-8 weeks (weighing 262.0–315.9 g, Charles River Laboratories Japan, Inc.), reared under temperature conditions of 23°C ± 2°C and relative humidity of 55% ± 15%, and then fed food (MF for rats, Oriental Yeast Co., Ltd.) and tap water ad libitum. Rats were used for study after preliminary breeding for 8 days or more.

### 2.3. Blood Radioactivity Measurement

The ^14^C-hyaluronic acid was administered orally at 25 mg/kg (*n* = 3) and intravenously at 10 mg/kg (*n* = 3) for plasma radioactivity measurement, which is an index of bioavailability. Consider the following:

(1) $$\begin{array}{c} \text{Bioavailability}\;\left(\%\right)=\frac{{\left[\text{AUC}\right]}_{\text{p}.\text{o}.}/{\text{DOSE}}_{\text{p}.\text{o}.}}{{\text{[AUC]}}_{\text{i.v.}}\text{/}{\text{DOSE}}_{\text{i.v.}}}\times 100, \end{array}$$

where [AUC]_p.o._ is oral administration [AUC]_i.v._/DOSE_i.v._ is oral dose, [AUC]_i.v._ is intravenous administration AUC, and DOSE_i.v._ is intravenous dose.

The test material dissolved in injectable distilled water was administered by single oral gavage to rats using a syringe with the oral probe into the stomach. Administered radioactivity was 2.04 MBq/kg body weight. Intravenous injection was performed in the femoral vein using a needle syringe. The administered radioactivity dose was 0.82 MBq/kg body weight. The transition of plasma ^14^C radioactivity was investigated by collecting blood over time from the tail vein of animals that received the single administration.

After collecting about 250 *μ*L of blood from the tail vein using a heparin-treated capillary tube (Terumo Corporation) at 5, 15, and 30 minutes and 1, 2, 4, 8, 24, 48, 72, 96, 120, 144, and 168 hours after administrating ^14^C-hyaluronic acid, followed by centrifugation (at 8060 ×g for 5 minutes at room temperature), the obtained 100 *μ*L of plasma was transferred to vials as radioactivity samples and dissolved by adding 2 mL of the tissue solubilizer Soluene-350 (Packard Instrument Company). After adding 10 mL of scintillator Hionic-Fluor (Packard Instrument Company) and standing at room temperature, the ^14^C-hyaluronic acid concentration was calculated by measuring radioactivity using the LSC (Liquid Scintillation Counter, 2700TR, Packard Instrument Company).

### 2.4. Measuring Excretion Rate in Urine, Feces, and Expired Air and Residual Rate in the Body

The animals that received ^14^C-hyaluronic acid in a single oral administration at a dose of 25 mg/kg (*n* = 3) were housed in metabolic cages, and the ^14^C-excretion rates in the urine, feces, and expired air were determined from the collected urine, feces, and expired air samples at predetermined times. After the last sampling, animals were killed under ether anesthesia to measure the residual rate in the body.

For measuring the urinary excretion rate, the cage was washed with a small amount of distilled water during 0 to 4, 4 to 8, 8 to 24, 24 to 48, 48 to 72, 72 to 96, 96 to 120, 120 to 144, and 144 to 168 hours after ^14^C-hyaluronic acid administration, and the water used for washing was put together with the urine, followed by further dilution to 100 mL with distilled water. After transferring the collected 1 mL of diluent to vials as radioactivity samples, 10 mL of scintillator HIONIC-FLUOR was added and urinary excretion rate was calculated from the radioactivity value measured using the LSC.

For measuring the fecal excretion rate, weighing the feces collected during 0–24, 24–48, 48–72, 72–96, 96–120, 120–144, and 144–168 hours after ^14^C-hyaluronic acid administration, adding distilled water, stirring to make homogeneous with the Polytron homogenizer (Kinematica Inc.), and further diluting to 100 mL with distilled water, the 0.2 mL was transferred to vials as radioactivity samples. After dissolving with heat by adding 2 mL of tissue solubilizer Soluene-350, adding 10 mL of scintillator Hionic-Fluor, and standing at room temperature, the feces excretion rate was calculated from the radioactivity value measured using the LSC.

For measuring the excretion rate in expired air, the excretion rate in expired air was calculated from the radioactivity value measured using the LSC after collecting expired air in two trap bottles linked in series that were filled with 200 mL of 20% monoethanolamine solution while venting air into a metabolic cage, transferring each 1 mL to vials as radioactivity samples and adding 10 mL of scintillator Hionic-Fluor.

For measuring the residual rate in the body, the residual rate in the body was calculated from the radioactivity value measured using the LSC after adding 500 mL of 0.5 mol/L sodium hydroxide solution and 50 mL of toluene to cadaver tissues, dissolving with heat to reflux, diluting to 900 mL with water, and stirring to make homogeneous, and the 0.5 mL was transferred to vial after adding 10 mL of scintillator Hionic-Fluor.

### 2.5. Whole Body Autoradiography

After animals that received ^14^C-hyaluronic acid in a single oral administration at a dose of 25 mg/kg were killed under ether paralysis at a predetermined time, radioactivity distributions and time-dependent changes to various tissues were examined by preparing whole body autoradiograms.

The animals were killed under ether anesthesia at 8, 24, and 96 hours after ^14^C-hyaluronic acid administration (*n* = 1 each), the hair coat was sheared immediately, and the nasal cavity and anus were blocked with 5% sodium carboxyl methyl cellulose (CMC-Na). The body was frozen in dry ice-acetone, separating the front and hind limbs and tail from the frozen cadaver, embedding with 5% CMC-Na on microtome stages, freezing in dry ice-acetone, fixing on cryomicrotome (PMV 450MP, Sweden PMV), and scraping off by sticking cryosections at a thickness 35 *μ*m on adhesive tape (no. 810, Sumitomo 3M Co., Ltd.) to be freeze dried. Predried sections were covered with a protective coat (4 *μ*m diagram foil, Mitsubishi Chemical Corp. polyester film Co., Ltd.) and tightly adhered to the imaging plate (Type-BAS SR2040, Fuji Photo Film Co., Ltd.) to be exposed for a certain period of time in a lead shield box. After exposure, whole body autoradiograms were prepared from radioactivity images recorded on imaging plates using BAS (Fujix BAS2500, Fuji Photo Film Co., Ltd.). Reading conditions for the imaging plate were a resolution of 50 *μ*m, gradation of 256, sensitivity of 30,000, and latitude of 5.

## 3. Results

### 3.1. Plasma Radioactivity Level

#### 3.1.1. Oral Administration Group

At the time of the single oral administration of ^14^C-hyaluronic acid at a dose of 25 mg/kg in male SD rats aged 7 to 8 weeks, plasma radioactivity rose slowly, *C*_max⁡_ (peak plasma radioactivity level) was 7.6 *μ*g eq/mL, *T*_max⁡_ (time at *C*_max⁡_) was 8 hours, *T*_1/2_ (24–168 h) (half-life) was 1.9 days, and AUC (0–∞) (area under the concentration-time curves of plasma) was 309 *μ*g of eq/mL/h (Table 1). ^14^C-hyaluronic acid migrated into the blood when orally administered.

#### 3.1.2. Intravenous Administration Group

At the time of single intravenous injection of ^14^C-hyaluronic acid at a dose of 10 mg/kg in male SD rats aged 7 to 8 weeks, the plasma radioactivity level was 233.0 *μ*g of eq/mL at 5 minutes after administration, which was the first measurement time, and *T*_1/2_ (5 minutes to 8 hours) decreased in 1.3 hours. *T*_1/2_ (24–168 hours) was 1.7 days, and AUC (0–∞) was 765 *μ*g of eq/mL/h (Table 1). Bioavailability calculated from the AUC of the oral administration group and intravenous administration group was 16%.

### 3.2. Excretion Rate in Urine, Feces, and Expired Air and Residual Rate in the Body

At the time of single oral administration of ^14^C-hyaluronic acid at a dose of 25 mg/kg in male SD rats aged 7 to 8 weeks, radioactivity was excreted in urine: 2.5% of the dose by 24 hours, 2.9% by 96 hours, and 3.0% by 168 hours after administration. In feces, ^14^C was excreted as follows: 7.8% of the dose by 24 hours, 11.6% by 96 hours, and 11.9% by 168 hours. In expired air, radioactivity was excreted as follows: 70.7% of the dose by 24 hours, 75.4% by 96 hours, and 76.5% by 168 hours. Total excretion rate in urine, feces, and expired air was 91.3% of the dose by 168 hours after administration, whereas 8.8% of the dose remained in the cadaver at this point of time (Table 2).

### 3.3. Whole Body Autoradiogram

**Figure 2:**
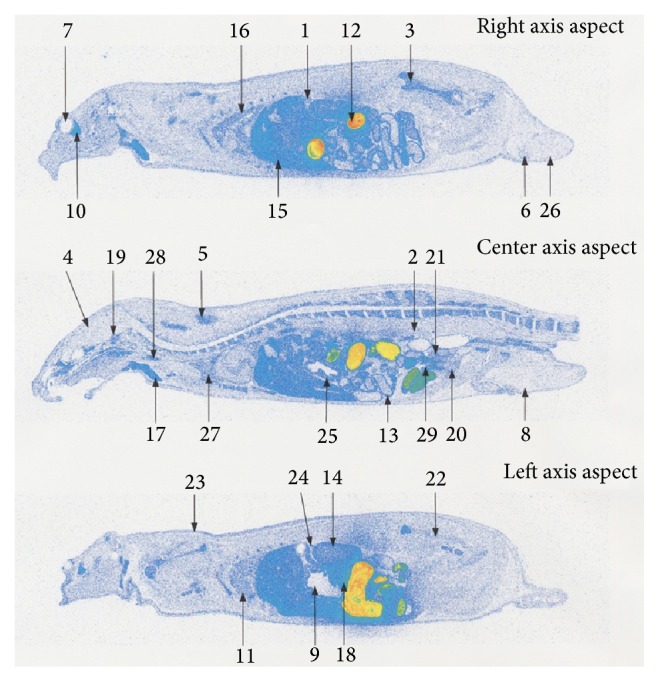
Whole body radioluminograms 8 hours after single oral administration of ^14^C hyaluronic acid to a rat (dose: 25 mg/kg). (1) Adrenal gland. (2) Blood. (3) Bone marrow. (4) Brain. (5) Brown fat. (6) Epididymis. (7) Eyeball. (8) Fat. (9) Gastric contents. (10) Harderian gland. (11) Heart. (12) Intestinal contents. (13) Intestine. (14) Kidney. (15) Liver. (16) Lung. (17) Mandibular gland. (18) Pancreas. (19) Pituitary gland. (20) Prostate gland. (21) Seminal vesicle. (22) Skeletal muscle. (23) Skin. (24) Spleen. (25) Stomach. (26) Testes. (27) Thymus. (28) Thyroid gland. (29) Urine in bladder.

**Figure 3:**
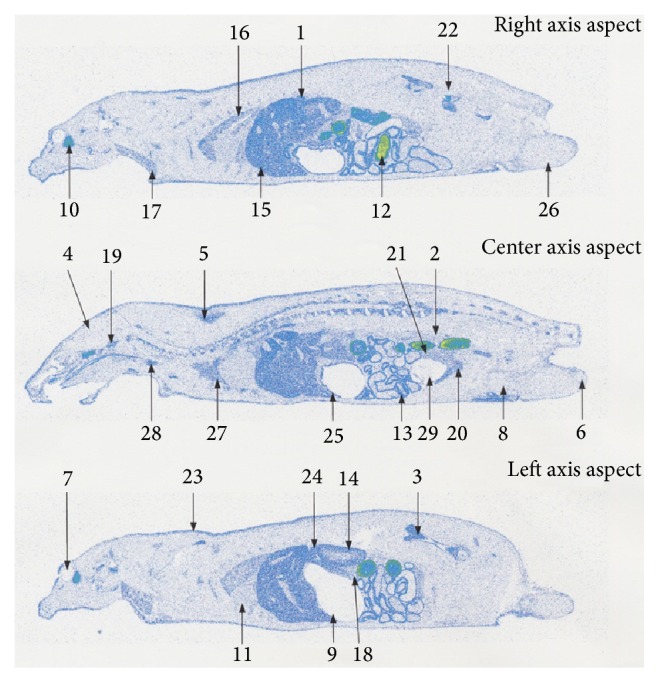
Whole body radioluminograms 24 hours after single oral administration of ^14^C hyaluronic acid to a rat (dose: 25 mg/kg). (1) Adrenal gland. (2) Blood. (3) Bone marrow. (4) Brain. (5) Brown fat. (6) Epididymis. (7) Eyeball. (8) Fat. (9) Gastric contents. (10) Harderian gland. (11) Heart. (12) Intestinal contents. (13) Intestine. (14) Kidney. (15) Liver. (16) Lung. (17) Mandibular gland. (18) Pancreas. (19) Pituitary gland. (20) Prostate gland. (21) Seminal vesicle. (22) Skeletal muscle. (23) Skin. (24) Spleen. (25) Stomach. (26) Testes. (27) Thymus. (28) Thyroid gland. (29) Urine in bladder.

**Figure 4:**
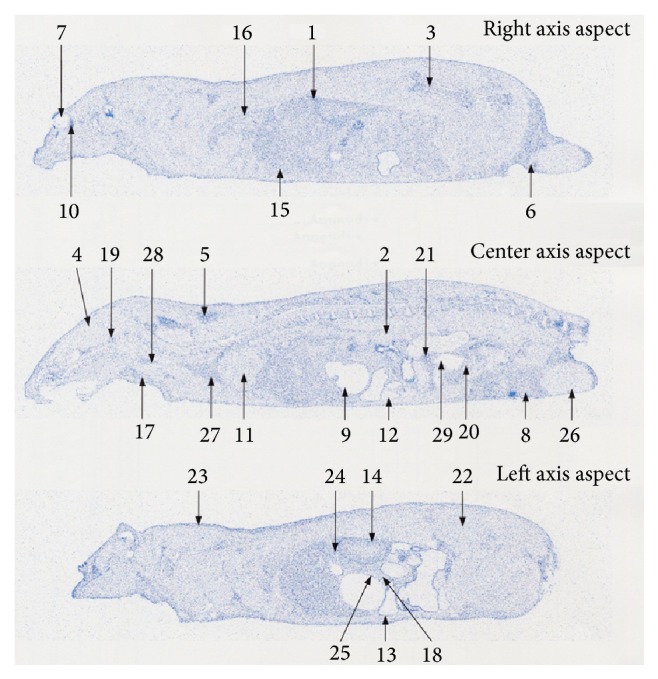
Whole body radioluminograms 96 hours after single oral administration of ^14^C hyaluronic acid to a rat (dose: 25 mg/kg). (1) Adrenal gland. (2) Blood. (3) Bone marrow. (4) Brain. (5) Brown fat. (6) Epididymis. (7) Eyeball. (8) Fat. (9) Gastric contents. (10) Harderian gland. (11) Heart. (12) Intestinal contents. (13) Intestine. (14) Kidney. (15) Liver. (16) Lung. (17) Mandibular gland. (18) Pancreas. (19) Pituitary gland. (20) Prostate gland. (21) Seminal vesicle. (22) Skeletal muscle. (23) Skin. (24) Spleen. (25) Stomach. (26) Testes. (27) Thymus. (28) Thyroid gland. (29) Urine in bladder.

At the time of single oral administration of ^14^C-hyaluronic acid at a dose of 25 mg/kg in male SD rats aged 7 to 8 weeks, ^14^C was detected in the skin as follows: 2.36 PSL/mm^2^ at 8 hours, 3.81 PSL/mm^2^ at 24 hours, and 1.98 PSL/mm^2^ at 96 hours after administration. ^14^C was detected in the blood as follows: 2.12 PSL/mm^2^ at 8 hours, 1.68 PSL/mm^2^ at 24 hours, and 0.84 PSL/mm^2^ at 96 hours after administration. The ^14^C level in the skin was similar to that in the blood at 8 hours after administration, whereas a higher ^14^C level was detected in the skin compared with the blood at 24 hours or more after administration (Table 3, Figures 2–4).

In the other tissues, the highest radioactivity was observed in the intestinal contents at 8 hours after administration; subsequently, readings in the pancreas, harderian gland, liver, and mandibular gland were high. The radioactivity in the following was higher than in the blood: the bowels, intravesical urine, spleen, kidney, thyroid gland, stomach, bone marrow, brown fat, lungs, seminal vesicle, adrenal gland, pituitary gland, thymus, heart, and prostate. Radioactivity at a similar level as in the blood was found in the epididymis, skeletal muscles, and the brain. The testes, white fat, gastric contents, and eyeballs showed lower radioactivity levels than in the blood (Table 3 and Figure 2). At 24 hours after administration, high radioactivity was observed in intestinal contents, and the harderian gland was subsequently high. Radioactivity in the following was higher than in the blood: bowels, bone marrow, adrenal gland, thyroid gland, liver, spleen, kidney, stomach, brown fat, pituitary gland, prostate, thymus, lungs, mandibular gland, and seminal vesicle. Radioactivity at a similar level to the blood was found in the pancreas and heart. The skeletal muscle, epididymis, brain, testes, white fat, eyeballs, intravesical urine, and gastric contents showed lower radioactivity levels than in the blood (Table 3 and Figure 3). At 96 hours after administration, all radioactivity dropped. Radioactivity in the following was higher than in the blood: harderian gland, seminal vesicle, adrenal gland, kidney, liver, brown fat, and bowels. Other tissues showed similar levels of radioactivity to the blood or lower levels of radioactivity than the blood (Table 3 and Figure 4).

## 4. Discussion

At the time of single oral administration of ^14^C-hyaluronic acid at a dose of 25 mg/kg in male SD rats aged 7 to 8 weeks, the plasma radioactivity level was less than the detection limit by 2 hours after administration. Four hours or more after that, ^14^C began to be detected and reached the maximum level at 8 hours after administration; therefore, hyaluronic acid was absorbed slowly from the digestive tract. At 8 hours or more after administration, *T*_1/2_  (24–168 hours) disappeared in 1.9 days, and this elimination half-life period was similar to that of the intravenous administration group at a dose of 10 mg/kg. Namely, hyaluronic acid was taken up by the body via the oral administration route.

For migration to the skin, hyaluronic acid reached the skin in this study though the molecular weight was unknown. The ^14^C derived from hyaluronic acid that reached the tissues via the oral route decreased over time, but there was similar or higher radioactivity distributed in the skin than in the blood even at 96 hours after administration; thus, this was considered a useful finding for the effect of hyaluronic acid on the skin.

Low-molecular-weight hyaluronic acid is reported to promote the growth of fibroblasts and to elevate hyaluronic acid synthesis [34, 35]. If orally administered hyaluronic acid should reach the skin in the form of low-molecular-weight hyaluronic acid, the phenomenon described above occurs and is considered to act on skin moisture improvement.

Hydroxyproline and ceramide, which are the components present in the skin as hyaluronic acid, are known to be transferred to the skin by ingestion. It is reported that 1.6% of ceramide was transferred to the skin 96 hours after ingestion [36]. Although the amount of hydroxyproline transferred to the skin by ingestion is unclear, it has been shown to reach the skin [37]. Thus it is well known that the components present in the skin reach the skin by ingestion. However, there is no other report on the transfer of macromolecular polysaccharide such as hyaluronic acid to the skin by ingestion than that of hyaluronic acid.

In regard to pharmacokinetics of ingested hyaluronic acid, it has been reported that low molecular hyaluronic acid passes through the intestine [30], endogenous hyaluronic acid is present in the blood, and hyaluronic acid is stable in the body [38, 39]. There is a report showing that hyaluronic acid labeled with ^99^Tc reached the skin [32]. In this report where movements of free ^99^Tc and ^99^Tc-hyaluronic acid to the skin were studied, free ^99^Tc reached the skin 30 min after administration and disappeared 120 min after administration, whereas ^99^Tc-hyaluronic acid reached the skin 4 hours after administration and remained in the skin even 72 hours after administration [32]. It was considered that, in the study, the compound reached the skin with maintaining the hyaluronic acid structure to some extent since ^99^Tc was administered as a chelate complex. In our study, ^14^C-radioactivity was detected in the skin 8 to 96 hours after administration. Therefore, it is possible that ingested ^14^C-hyaluronic acid reached the skin as a form of hyaluronic acid. However, the existence form of ^14^C in the skin was not directly analyzed in the current study. Thus, the identification of substance containing ^14^C which reached the skin and the involvement of this substance in the skin moisture retention effect needs to be addressed in the future.

For metabolism, a part of the hyaluronic acid used in body tissues is physiologically subject to keratinization and subsequent desquamation in the epidermis [40], and the rest is subject to degradation by hyaluronidase [41, 42] or fragmentation by oxygen radicals in each tissue [43–47]. Partially degraded hyaluronic acid is reported to enter local lymph nodes through lymphatic vessels following a fragmentation process and subsequently enters the blood circulation system, going through final hydrolysis in the liver [48, 49] and then eliminated from the body. This study examined extracorporeal excretion at the time of single oral administration of ^14^C-hyaluronic acid at a dose of 25 mg/kg in male SD rats aged 7 to 8 weeks; consequently, the main excretion route was via expired air, resulting in 76.5% of the dose being excreted by 168 hours after administration. Urinary and fecal excretion rates were 11.9% and 3.0% of the dose, respectively, by 168 hours after administration, whereas radioactivity remained at 8.8% of the dose in the body at this time.

Approximately 90% of ^14^C-hyaluronic acid was absorbed from the digestive tract and used as an energy source or a structural constituent of the body. A total of 16% bioavailability determined from AUC was a low value compared with the 90% absorption rate. Typically, high-molecular polysaccharides, such as starch, are absorbed from the small intestine and then carried to the liver via the hepatic portal vein from the capillary bed and are known to be metabolized in the liver. Orally ingested hyaluronic acid is mostly metabolized in the liver and degraded into carbon dioxide (first-pass effect); bioavailability was low because hyaluronic acid was eliminated as expired air from the lungs before entering whole-body blood circulation [50].

In recent years, an aging society is advancing in Japan, USA, and Europe. Hyaluronic acid in the body decreases with age, and accordingly, the hyaluronic acid content in the skin of 75-year-old elderly persons is lower than one-quarter compared with that of a 19-year-old youth [51]. Thus, the need in food applications for hyaluronic acid is increasing to supplement deficient hyaluronic acid. This study can support the finding that hyaluronic acid reaches the skin and that hyaluronic acid does not accumulate excessively. We hope this will lead to QOL improvement and relief for the elderly and people suffering from dry skin.

## 5. Conclusions

This study's results have shown the possibility that orally ingested ^14^C-hyaluronic acid was taken up into the body and migrated into the skin. Further study is required, including the molecular weight in the blood of orally ingested hyaluronic acid and confirmation by skin tissue extraction. Also, after orally ingested hyaluronic acid was used by tissues, 90% or more was metabolized and eliminated in expired air and urine, suggesting that there was no excessive accumulation in the body.

## Figures and Tables

**Table 1:** Radioactivity concentration in plasma after single oral or intravenous administration of ^14^C hyaluronic acid to rats (dose: p.o.; 25 mg/kg, IV; 10 mg/kg).

Time	Radioactivity concentration	
	(*μ*g eq. of hyaluronic acid/mL)	
	p.o.	i.v.
5 min	N.D.	233.0 ± 22.1
15	N.D.	222.5 ± 15.7
30	N.D.	211.7 ± 8.4
1 hr	N.D.	180.1 ± 2.6
2	N.D.	123.1 ± 2.0
4	1.1 ± 0.8	47.8 ± 9.4
8	7.6 ± 0.6	3.6 ± 0.3
24	3.5 ± 0.3	1.7 ± 0.1
48	2.0 ± 0.1	0.9 ± 0.1
72	1.3 ± 0.1	0.6 ± 0.1
96	0.9 ± 0.0	0.4 ± 0.1
120	0.7 ± 0.1	0.3 ± 0.0
144	0.5 ± 0.0	0.2 ± 0.0
168	0.4 ± 0.1	0.1 ± 0.1

Detection limit	0.1	0.1

*T*_max⁡_ (hr)	8 ± 0	—

*C*_max⁡_ (*μ*g eq./mL)	7.6 ± 0.6	—

*C*_0_ (*μ*g eq./mL)	—	238.4 ± 25.5

*T*_1/2_		
(5 min–8 hr) (hr)	—	1.3 ± 0.1
(24–168 hr) (day)	1.9 ± 0.1	1.7 ± 0.1

AUC (*μ*g eq.*·*hr/mL)		
(0–168 hr)	284 ± 16	757 ± 37
(0–*∞*)	309 ± 20	765 ± 40

Data are expressed as the mean values ± S.D. of three animals.

N.D.: not detected.

—: not determined.

**Table 2:** Cumulative excretion of radioactivity in urine, feces, and expired air as ^14^CO_2_ after single oral administration of  ^14^C hyaluronic acid to rats (dose: 25 mg/kg).

Time (hr)	Excretion of radioactivity (% of dose)			
	Urine	Feces	Expried air	Total
0–4	0.0 ± 0.0	—	1.5 ± 1.2	—
8	1.0 ± 0.3	—	45.9 ± 1.9	—
24	2.5 ± 0.3	7.8 ± 1.6	70.7 ± 1.2	81.1 ± 1.5
48	2.7 ± 0.2	11.0 ± 1.0	73.4 ± 1.3	87.0 ± 0.9
72	2.8 ± 0.2	11.4 ± 1.0	74.7 ± 1.5	88.8 ± 0.9
96	2.9 ± 0.2	11.6 ± 1.0	75.4 ± 1.5	89.9 ± 1.0
120	2.9 ± 0.2	11.7 ± 1.0	75.8 ± 1.5	90.4 ± 1.0
144	2.9 ± 0.3	11.8 ± 1.0	76.2 ± 1.5	90.9 ± 1.0
168	3.0 ± 0.2	11.9 ± 1.0	76.5 ± 1.6	91.3 ± 1.0

Carcass (168 hr)				8.8 ± 0.6

Data are expressed as the mean values ± S.D. of three animals.

—: not determined.

**Table 3:** The distribution of radioactivity in selected organs and tissues as [(photostimulated luminescence-background)/area] after single oral administration of ^14^C hyaluronic acid to rats at a dose of 25 mg/kg.

No.	Tssue	Distribution of radio activity (PSL/mm^2^)		
		8 hr	24 hr	96 hr
1	Adrenal gland	3.74	4.94	2.11
2	Blood	2.12	1.68	0.84
3	Bone marrow	4.88	5.00	1.31
4	Brain	1.20	0.88	0.54
5	Brown fat	4.66	3.68	2.16
6	Epididymis	1.76	1.03	0.69
7	Eyeball	0.57	0.38	0.26
8	Fat	0.83	0.83	1.19
9	Gastric contents	0.59	0.10	0.04
10	Harderian gland	12.27	18.75	2.72
11	Heart	3.09	1.27	0.88
12	Intestinal contents	710.02	86.68	0.28
13	Intestine	6.60	8.16	2.09
14	Kidney	5.79	3.93	1.51
15	Liver	9.22	4.48	1.42
16	Lung	4.10	2.93	1.21
17	Mandibular gland	7.49	2.65	1.31
18	Pancreas	17.45	2.10	1.02
19	Pituitary gland	3.67	3.58	1.60
20	Prostate gland	2.99	3.13	1.13
21	Seminal vesicle	4.03	2.43	2.34
22	Skeletal muscle	1.68	1.04	0.83
23	Skin	2.36	3.81	1.98
24	Spleen	6.07	4.43	1.84
25	Stomach	5.34	3.75	1.30
26	Testes	0.88	0.86	0.57
27	Thymus	3.16	2.99	1.45
28	Thyroid gland	5.45	4.54	1.52
29	Urine in bladder	6.20	0.34	0.06
